# Abnormal Skeletal Muscle Regeneration plus Mild Alterations in Mature Fiber Type Specification in *Fktn*-Deficient Dystroglycanopathy Muscular Dystrophy Mice

**DOI:** 10.1371/journal.pone.0147049

**Published:** 2016-01-11

**Authors:** Steven J. Foltz, Jill N. Modi, Garrett A. Melick, Marin I. Abousaud, Junna Luan, Marisa J. Fortunato, Aaron M. Beedle

**Affiliations:** Department of Pharmaceutical and Biomedical Sciences, University of Georgia, Athens, Georgia, United States of America; Ohio State University Medical Center, UNITED STATES

## Abstract

Glycosylated α-dystroglycan provides an essential link between extracellular matrix proteins, like laminin, and the cellular cytoskeleton via the dystrophin-glycoprotein complex. In secondary dystroglycanopathy muscular dystrophy, glycosylation abnormalities disrupt a complex *O*-mannose glycan necessary for muscle structural integrity and signaling. *Fktn*-deficient dystroglycanopathy mice develop moderate to severe muscular dystrophy with skeletal muscle developmental and/or regeneration defects. To gain insight into the role of glycosylated α-dystroglycan in these processes, we performed muscle fiber typing in young (2, 4 and 8 week old) and regenerated muscle. In mice with *Fktn* disruption during skeletal muscle specification (Myf5/*Fktn* KO), newly regenerated fibers (embryonic myosin heavy chain positive) peaked at 4 weeks old, while total regenerated fibers (centrally nucleated) were highest at 8 weeks old in tibialis anterior (TA) and iliopsoas, indicating peak degeneration/regeneration activity around 4 weeks of age. In contrast, mature fiber type specification at 2, 4 and 8 weeks old was relatively unchanged. Fourteen days after necrotic toxin-induced injury, there was a divergence in muscle fiber types between Myf5/*Fktn* KO (skeletal-muscle specific) and whole animal knockout induced with tamoxifen post-development (Tam/*Fktn* KO) despite equivalent time after gene deletion. Notably, Tam/*Fktn* KO retained higher levels of embryonic myosin heavy chain expression after injury, suggesting a delay or abnormality in differentiation programs. In mature fiber type specification post-injury, there were significant interactions between genotype and toxin parameters for type 1, 2a, and 2x fibers, and a difference between Myf5/*Fktn* and Tam/*Fktn* study groups in type 2b fibers. These data suggest that functionally glycosylated α-dystroglycan has a unique role in muscle regeneration and may influence fiber type specification post-injury.

## Introduction

The dystrophin-glycoprotein complex (DGC), including dystrophin, dystroglycan, sarcoglycans, sarcospan and additional intracellular scaffold and signaling molecules, provides an important connection from the intracellular actin cytoskeleton to the extracellular matrix in skeletal muscle and other tissues [1–3]. Extracellular α- and transmembrane β-dystroglycan (αDG, βDG) are crucial to this link as unique *O*-mannose glycan structures on αDG bind directly to laminin, agrin, neurexin, and perlecan in the basement membrane, conveying structural and signaling integrity through αDG to βDG and dystrophin [4–11]. Functional loss of this axis causes various forms of muscular dystrophy including X-linked Duchenne and Becker muscular dystrophies (dystrophin), and a number of autosomal recessive congenital and limb girdle muscular dystrophies [12]. Secondary dystroglycanopathy, a disease with normal dystroglycan and DGC protein expression but abnormal αDG glycosylation, is emerging as a large and diverse class of autosomal recessive muscular dystrophies caused by mutations in one of 16 known genes involved in the *O*-mannose glycosylation of αDG [for review, see [13]]. This class of muscular dystrophies encompasses a broader range of phenotypes (severe congenital to mild late onset) than other DGC-related diseases, the varied pathomechanisms downstream of the loss of αDG-matrix binding are poorly delineated, and there are no validated therapies for patients.

Recent work by our group and others demonstrates a role for contraction-induced sarcolemmal damage in the dystrophic progression of dystroglycanopathy, but also indicates a role for αDG in skeletal muscle development and/or regeneration processes in more severe disease phenotypes [14–18]. Both developmental and regeneration processes influence the distribution of muscle fiber types as marked by the expression of various myosin heavy chain proteins. Type 1 fibers (expressing *MYH7*) confer a slow, fatigue-resistant muscle profile, while type 2 fibers are fast with variable fatigue: type 2a (*MYH2*), type 2x (*MYH1*), and type 2b (*MYH4*) isoforms have fast/oxidative, fast/intermediate and fast/glycolytic profiles, respectively, although humans do not express the type 2b isoform [for review see [19]]. Postnatally, immature isoforms are lost and fast isoforms emerge due to a combination of signals including increased load on the muscle, maturation of the neuromuscular junction, and a rise in thyroid hormone [19]. Slow muscle fiber types may persist from the embryo or arise from a conversion of type 2a fibers to type 1 around 4 weeks of age [20].

Fiber death after a necrotic incident stimulates activation and proliferation of muscle resident stem cells, “satellite cells” [for review, see [21]]. Resulting myoblasts (expressing embryonic heavy chain) can fuse to one another and remaining mature fibers to repopulate the muscle compartment. Post-regeneration fusion typically leads to fiber type specification that is dependent on the stimulation frequency from the innervating motor neuron with low frequency stimulation driving type 1 differentiation and high frequency or aneural fibers becoming fast [19]. Transition between fiber types is a common event in skeletal muscle and often reflects an adaptive response of muscles to use or disuse; because myosin protein turnover is slow, muscles experiencing fiber type transformation frequently harbor hybrid fibers with mixed myosin isotypes (e.g. 2a/x, 2b/x)[19]. As the expression profile of distinct skeletal muscle fiber types convey specific contractile, metabolic, and fatigue properties, variation in fiber type specification during development or regeneration in muscular dystrophy influences patient phenotype. In fact, variation in fiber type proportions and/or fiber type selective susceptibility to disease has been reported in a number of muscular dystrophies, including Duchenne muscular dystrophy (DMD), myotonic dystrophy, facioscapulohumeral and oculopharyngeal muscular dystrophies [e.g. [22–25]]. While fiber types have not been analyzed in any dystroglycanopathy model, muscle force measurements from the extensor digitorum longus (EDL) and soleus of *Large*^myd/myd^ mice suggested an increased susceptibility of type 2 fibers to contraction-induced injury in dystroglycanopathy muscle [26].

To better understand the abnormal processes during skeletal muscle development and regeneration in dystroglycanopathy muscular dystrophy, we performed fiber isotype analysis in two mouse models with conditional knockout of dystroglycanopathy gene *Fktn*. In the Myf5-cre/*Fktn* knockout (Myf5/*Fktn* KO), gene disruption at embryonic day 8 initiates a dystroglycan glycosylation defect during skeletal muscle development, affecting downstream satellite cells and muscle fibers [15]. In the whole animal inducible knockout, Cre-ER is expressed in all cells, but only translocates to the nucleus for gene excision when tamoxifen is present (tamoxifen-cre/*Fktn* KO mice, Tam/*Fktn* KO). In these Tam/*Fktn* KO inducible mice, *Fktn* gene knockout was induced in skeletal muscle (and all other tissue types) post-development (15). Our data indicate changes in the regeneration process and mild changes to fiber type differentiation post-injury, suggesting that functional αDG plays a role in these processes that may contribute to disease progression and phenotype.

## Materials and Methods

### Ethics Statement

All mouse procedures were approved by the University of Georgia Institutional Animal Care and Use Committee (AUP A2010 08–163, A2013 07–016). All efforts were made to minimize animal suffering.

### Mice

Mice were maintained on a 12:12 light:dark cycle with standard husbandry and a supplement of wet food pellets on the cage floor 2 to 4 times per week. Myf5-cre/*Fktn* and whole animal inducible Tam-cre/*Fktn* conditional exon 2 knockout mice have been described previously, were a kind gift from Dr. Kevin Campbell (U. Iowa) and correspond to Jackson Laboratory strains #007893, #004682, and #019097 [15]. Myf5-cre/*Fktn* knockouts (Myf5/*Fktn* KO; Myf5^+/cre^;*Fktn*^L/L^) were bred from Myf5^+/cre^;*Fktn*^L/+^ x Myf5^+/+^;*Fktn*^L/L^ or Myf5^+/cre^;*Fktn*^L/+^ x Myf5^+/+^;*Fktn*^L/+^ pairs. Whole animal CAG-creERT2 (Cre-ER) tamoxifen inducible *Fktn* knockout mice (Tg^+/Cre-ER^;*Fktn*^L/-^; Tam/*Fktn* KO) were bred from Tg^+/Cre-ER^;*Fktn*^+/-^ x *Fktn*^L/L^ pairs so induction of Cre recombination was only necessary at one allele to induce homozygous exon 2 deletion. To induce recombination for gene knockout in Tam/*Fktn* KO mice, tamoxifen (Tam; Sigma, St. Louis, MO; or Cayman Chemical, Ann Arbor, MI) was dissolved in ethanol and diluted with sunflower oil (Sigma) to 100 mg/ml for delivery by oral gavage at 0.4 mg/g. Mice received the first round of Tam-treatment on two non-consecutive days (day 1 and 3) at 6 to 8 weeks of age and a second round of Tam-treatment 8 weeks later at 1 day pre- and 1 day post-toxin treatment. All littermate control mice were Tam-treated at the same time as their inducible KO littermates; all of the following genotypes were used for Tam/Fktn LC mice as we previously demonstrated that heterozygotes and *Fktn* floxed mice have no phenotype: Tg^+/Cre-ER^,Fktn^L/+^; Tg^+/+^, Fktn^L/-^ or Tg^+/+^, Fktn^L/+^ [15].

A total of 26 animals were used in the analysis of 2, 4, and 8 week old (wko) iliopsoas and TA muscles: 2 wko Myf5/*Fktn* KO and LC, n = 4 each; 4 wko Myf5/*Fktn* KO and LC, n = 4 each; (except Myf5/*Fktn* LC iliopsoas, n = 3); 8 wko Myf5/*Fktn* KO and LC, n = 5 each. 27 mice were used for the Myf5/*Fktn* and Tam/*Fktn* regeneration study, and the CTX-treated and contralateral saline-treated muscles were collected from each mouse: Myf5/*Fktn* LC, n = 5; Myf5/*Fktn* KO, n = 7; Tam/*Fktn* LC, n = 7; Tam/*Fktn* KO, n = 8.

### Cardiotoxin-induced regeneration

On the day of cardiotoxin injection, mice were anesthetized by isoflurane, lower hindlimbs were shaved, and mice were given an analgesic dose of meloxicam 1 mg/kg (VWR, Radnor, PA) by subcutaneous injection. Cardiotoxin (Sigma), at 10 μM in 0.9% sterile saline, was delivered in 25 μL intramuscular injections longitudinal into the tibialis anterior (TA, with potential involvement of the extensor digitorum longus, EDL). Identical injection of sterile 0.9% NaCl was completed for the left hindlimb (saline vehicle control). All toxin injections were performed by the same experimenter (AMB) for consistency across the dataset. Mice were euthanized 14 days post-injection and muscles were dissected for cryopreservation according to standard protocols [15,27]. Note, for this study, the TA and EDL were dissected out and analyzed as a single unit.

### Immunofluorescence and microscopy

The iliopsoas or TA/EDL were cryosectioned to a depth of 1 mm from the proximal side, then 7 μm sections were cut and mounted on glass slides for histological and fiber type analysis. Sections analyzed were derived from a comparable 300 μm zone of Ilio or TA, respectively, across all samples, accounting for minor variations in muscle dissection and a subset of samples that required re-sectioning. Immunofluorescent staining for myosin isoforms was performed using mouse monoclonal myosin heavy chain antibodies F1.652 (embryonic), BF-35 (all but 2x), BF-F3 (type 2b), SC-71 (type 2a), and BA-D5 (type 1) (Developmental Studies Hybridoma Bank, University of Iowa, Iowa City, IA) at 1:20–1:40 dilution. Samples were co-stained for nuclei (DAPI; Life Technologies, Grand Island, NY) and membrane/basement membrane counterstain by perlecan (heparin sulfate proteoglycan; EMD Millipore, Darmstadt, Germany) or αDGct rabbit monoclonal 5–2 [28] or related hybridomas 29–10 or 45–4, not previously reported. Neuromuscular junctions were analyzed using rabbit monoclonal antibody D35E4 against presynaptic marker synaptophysin (Cell Signaling Technology, Danvers, MA) and Alexa 488-coupled bungarotoxin for detection of nicotinic acetylcholine receptors at the postsynaptic endplate (Life Technologies). Muscle sections were blocked in 5% donkey serum in PBS for 30 min, incubated in primary antibody in 5% donkey serum at 4°C overnight, washed 3 x 5 min, incubated in secondary antibody (AlexaFluor A546 anti-mouse IgG1, IgG2b, or IgM and AlexaFluor A488 anti-rabbit or rat IgG; Life Technologies) with or without A488-bungarotoxin at 1:500 in 5% donkey serum for 30 min at room temperature, washed 3 x 5 min and mounted with PermaFluor (ThermoScientific, Waltham, MA). Detection of glycosylated αDG by indirect immunofluorescence with IIH6 antibody has been described previously [15]. Tissues were viewed by 20X objective on an inverted epifluorescent microscope (Olympus, Center Valley, PA) and images were captured using a DP-72 camera and CellSens software (Olympus).

For image analyses, a series of overlapping images crossing the entire muscle section were taken and compiled into a section map in Photoshop (Adobe). Compiled maps were analyzed by blinded observers in Image Pro Express (Media Cybernetics, Rockville, MD) for fiber counts and whole section area; a subset (all knockouts at 14 d post-CTX) was also analyzed for single fiber area of embryonic myosin heavy chain-positive (eMHC) fibers. Whole section areas from compiled maps were compared for serial sections of the same muscle and the most representative section map for each muscle was selected for total fiber counting. Mean Ilio section areas were: Myf5/*Fktn* 2 wko LC 0.72 ± 0.21 mm^2^; KO 0.56 ± 0.18 mm^2^; 4 wko LC 1.08 ± 0.20 mm^2^; KO 0.96 ± 0.13 mm^2^; 8 wko LC 1.73 ± 0.35 mm^2^; KO 1.21 ± 0.29 mm^2^; two-way ANOVA age, p < 0.05. Mean TA/EDL section areas were: Myf5/*Fktn* 2 wko LC 1.20 ± 0.05 mm^2^; KO 1.89 ± 0.29 mm^2^; 4 wko LC 2.72 ± 0.39 mm^2^; KO 1.95 ± 0.12 mm^2^; 8 wko LC 5.13 ± 0.64 mm^2^; KO 3.25 ± 0.46 mm^2^; two-way ANOVA genotype*age, p < 0.05; age, p < 0.0001. Any serial section that deviated by ≥ 15% from the counted map was also counted separately for total fibers. Total fiber counts ranged from 246 to 1716 in the iliopsoas and from 920 to 3795 in the TA of 2, 4, and 8 wko Myf5/*Fktn* mice; total fibers ranged from 1157 to 7483 in TAs of Myf5/*Fktn* or Tam/*Fktn* mice enrolled in the CTX study. Positive fibers (centrally nucleated [CN], eMHC) were counted by manual tag and divided by the number of total fibers in the section map x 100. Proportions of mature fiber types (MHC type 1, 2a, or 2b) were analyzed as the percentage of a given fiber type per total mature fibers (total fibers within a muscle section less eMHC expressing fibers) for each sample. Proportions of MHC type 2x fibers were determined as the percentage of fibers not stained by the MHC “all but 2X” antibody, which detects all MHC isoforms except type 2x, per total mature fibers for each sample.

### Graphs and Statistics

Fiber-type and central nucleation data are plotted as scatterplots with each data point representing analysis from one study animal; mean and standard error are shown. In text data references are also reported as mean with standard error. Data are analyzed by two-way ANOVA with Bonferroni’s post-test, which compares each row by all columns and each column by all rows (Prism 5, GraphPad, La Jolla, CA). Because separate littermate and knockout data are used from the two strains (Myf5/*Fktn* and Tam/*Fktn*) in fiber typing post-injury, we performed two-way ANOVA across all genotypes to facilitate comparisons between the two different types of knockout mice (e.g. Myf5/Fktn KO injured vs Tam/Fktn KO injured). Post-tests from this analysis of all genotype groups are denoted on figures using asterisks. We also performed two-way ANOVA on each strain group independently for the toxin-induced regeneration experiments (Myf5/*Fktn* only and Tam/*Fktn* only) to enable further interpretation of significance detected in the “all genotypes” analysis. Two-way ANOVA interaction (Genotype*Toxin or Genotype*Age) p values are reported on each figure; individual group effects (Genotype, Age, or Toxin) are only reported if the interaction p value is not significant. eMHC-positive areas are plotted as proportion of fibers within a given size range (bin) per total number of eMHC-expressing fibers. Optimal bin sizes for eMHC-positive fiber areas were determined previously [15]. Fiber-size distributions were non-normal (D’agostino & Pearson omnibus normality test failed), so data were analyzed by a two-tailed Mann-Whitney test (Prism 5, GraphPad, La Jolla, CA). p < 0.05 is considered statistically significant in all tests. *, p < 0.05; **, p < 0.01; ***, p < 0.001.

## Results

### Dystrophy progressively increases in *Fktn*-deficient muscle

**Fig 1:**
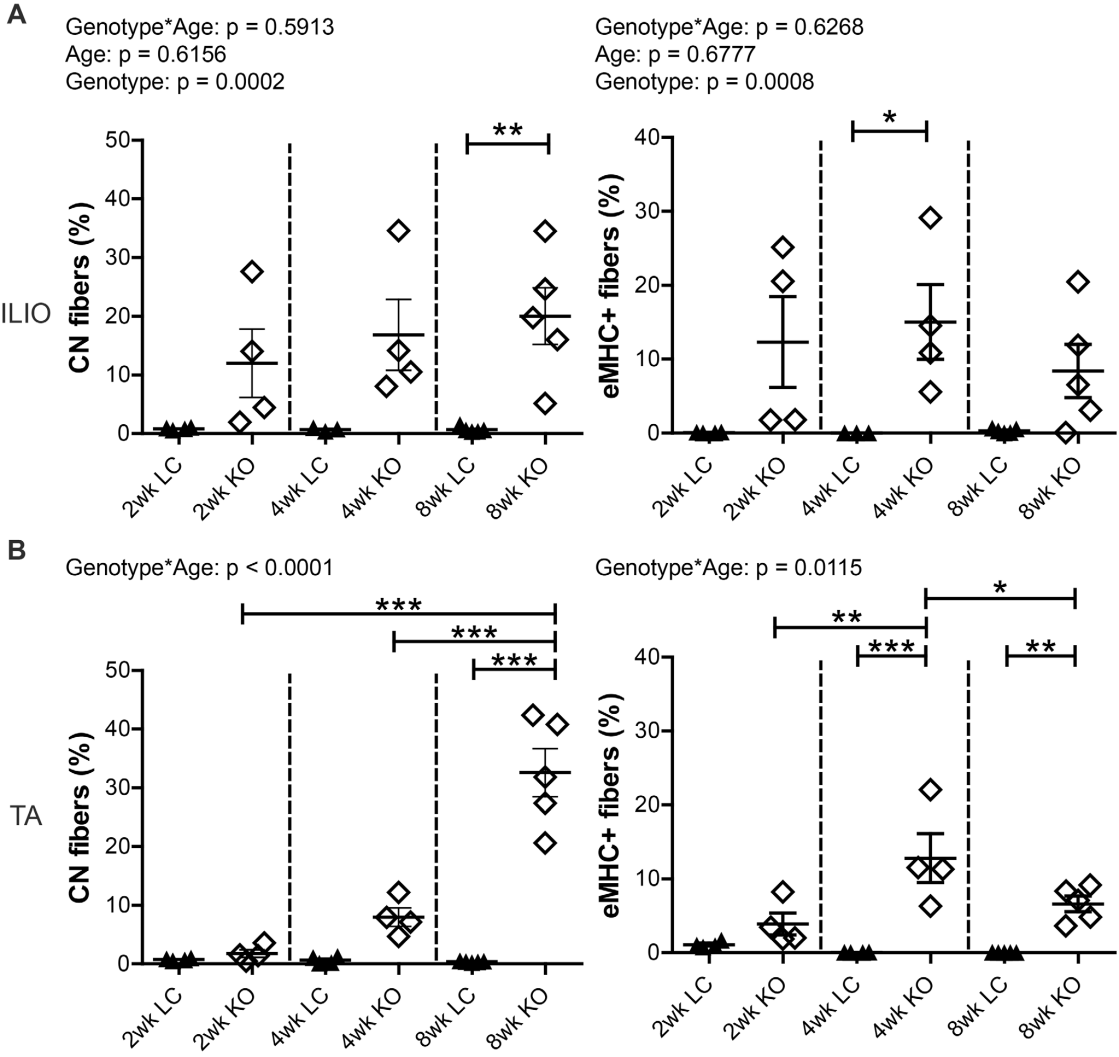
Dystrophy is prevalent and progressive in Myf5/*Fktn* KO muscle. Muscle regeneration after fiber necrosis as measured historically by central nucleation (CN, left) or transiently by embryonic myosin heavy chain expression (eMHC, right) in (A) iliopsoas or (B) TA muscle of 2, 4, and 8 wko Myf5/*Fktn*-deficient (KO) or control (LC) mice. *, p<0.05; **, p<0.01; ***, p<0.001. Two-way ANOVA with Bonferroni’s post-test. TA, n = 4 mice per 2 and 4 wko group, n = 5 per 8 wko group; Ilio, n = 3–4 per 2 and 4 wko group, n = 5 per 8 wko group.

Sarcolemmal attachment to the extracellular matrix through specific glycan structures on αDG is critical for muscle protection against contraction-induced damage [14]. Accordingly, loss of *Fktn* or other genes involved in processing the *O*-Mannose glycan on αDG renders the sarcolemma vulnerable to contraction-induced damage and myonecrosis [15,16,26,29,30]. To determine postnatal disease onset, we evaluated muscle regeneration in Myf5/*Fktn* KO muscle by the percentage of eMHC-expressing and centrally nucleated (CN) fibers in the iliopsoas and TA (with EDL) at 2, 4, and 8 weeks old (wko). CN was significantly increased in both iliopsoas and TA muscles of Myf5/*Fktn* KO mice, with a significant interaction between genotype and age in the TA, but not the iliopsoas (Fig 1A and 1B left). Post-tests identified elevated CN, a long-term marker of muscle regeneration, in 8 wko Myf5/*Fktn* KO TA (8 wko KO CN = 32.6 ± 4.1%) and iliopsoas (8 wko KO CN = 20.0 ± 4.8%), but a clear age-dependent increase in knockout muscle CN was only present for the TA muscle, due to an earlier onset of pathology in iliopsoas of some KO mice. In contrast, the proportion of eMHC-expressing fibers in both muscles was highly variable. However, there was a significant interaction between genotype and age (genotype*age) in the TA only; with increased eMHC-positive fibers in Myf5/*Fktn* KOs at 4 and 8 wko, reaching maximum at 4 weeks (4 wko KO eMHC = 12.8 ± 3.3%; Fig 1A and 1B right). In the Ilio, eMHC regenerating fibers were increased in Myf5/*Fktn* KO mice, with post-test significance at 4 weeks (4 wko KO eMHC = 15.0 ± 5.0%). Because eMHC is only temporarily expressed in regenerating muscle, it represents a “snapshot” of current regeneration at the time of analysis. Consequently, proportions of eMHC-expressing fibers are more variable and do not necessarily increase over time; CN, however, may persist for several months following a regeneration event, making it a suitable tool for examining the progression of disease. Thus, these data indicate that, on average, 20–30% of all muscle fibers have regenerated by 8 wko, confirming the development and progression of dystrophy in *Fktn*-deficient muscle and indicating a period of highly active muscle degeneration/regeneration peaking at 4 weeks and subsiding at 8 weeks.

### Fiber type specification shows age-dependent differences

**Fig 2:**
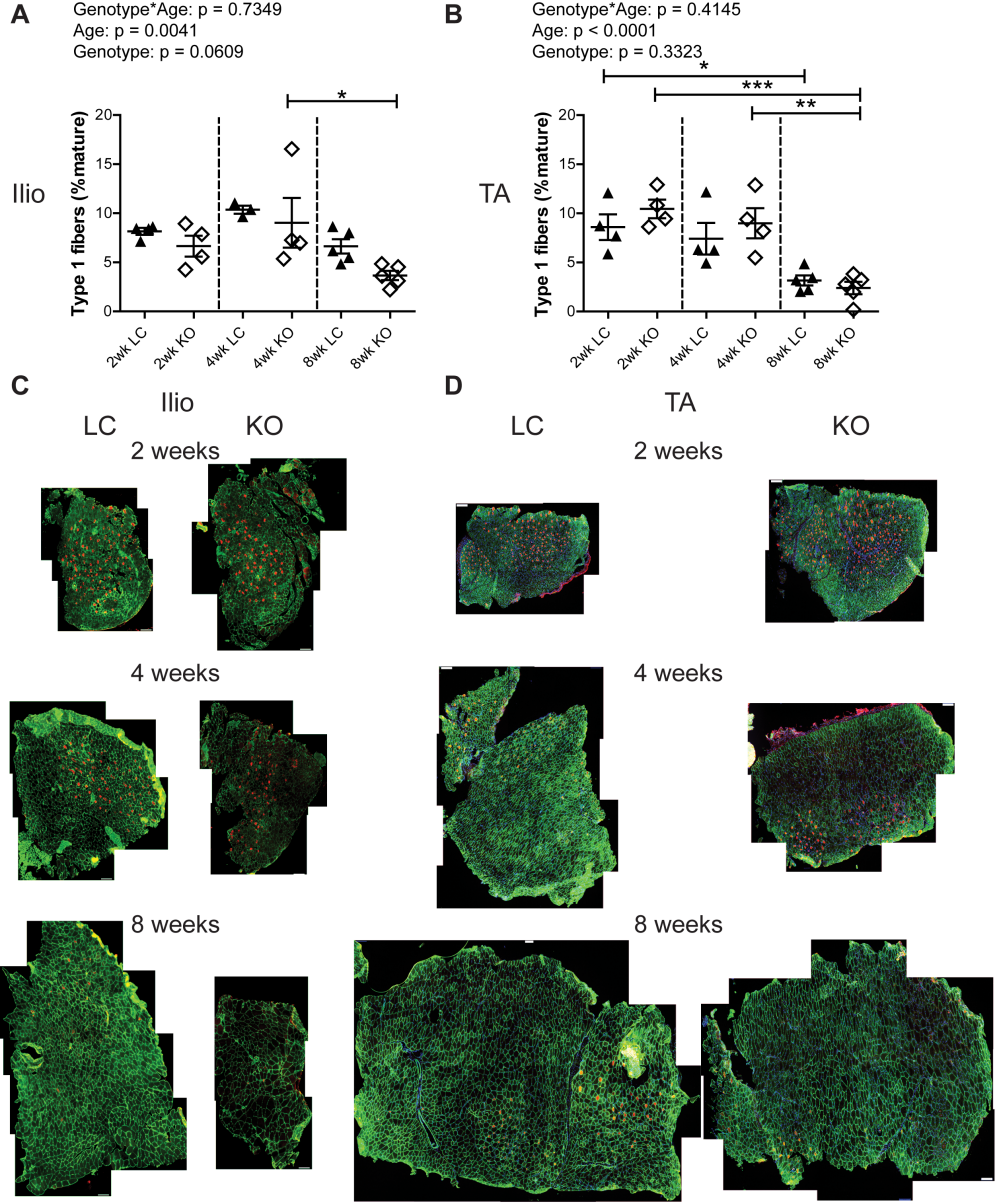
Frequency of type 1 oxidative fibers decreases with age in iliopsoas and TA. Oxidative type 1 fibers in (A) iliopsoas and (B) TA of 2, 4, and 8 wko Myf5/*Fktn*-deficient (KO) and control (LC) mice. *, p<0.05; **, p<0.01; ***, p<0.001, two-way ANOVA with Bonferroni’s post-test. Whole tissue (C) iliopsoas and (D) TA maps of sections stained with anti-myosin heavy chain type 1 antibody (red), with basement membrane perlecan or sarcolemmal αDG core protein (green) and nuclear (blue) counterstains. Scale bar = 100 μm. n = 4 for all 2 and 4 wko measurements (except Ilio LC, n = 3); n = 5 for all 8 wko measurements.

**Fig 3:**
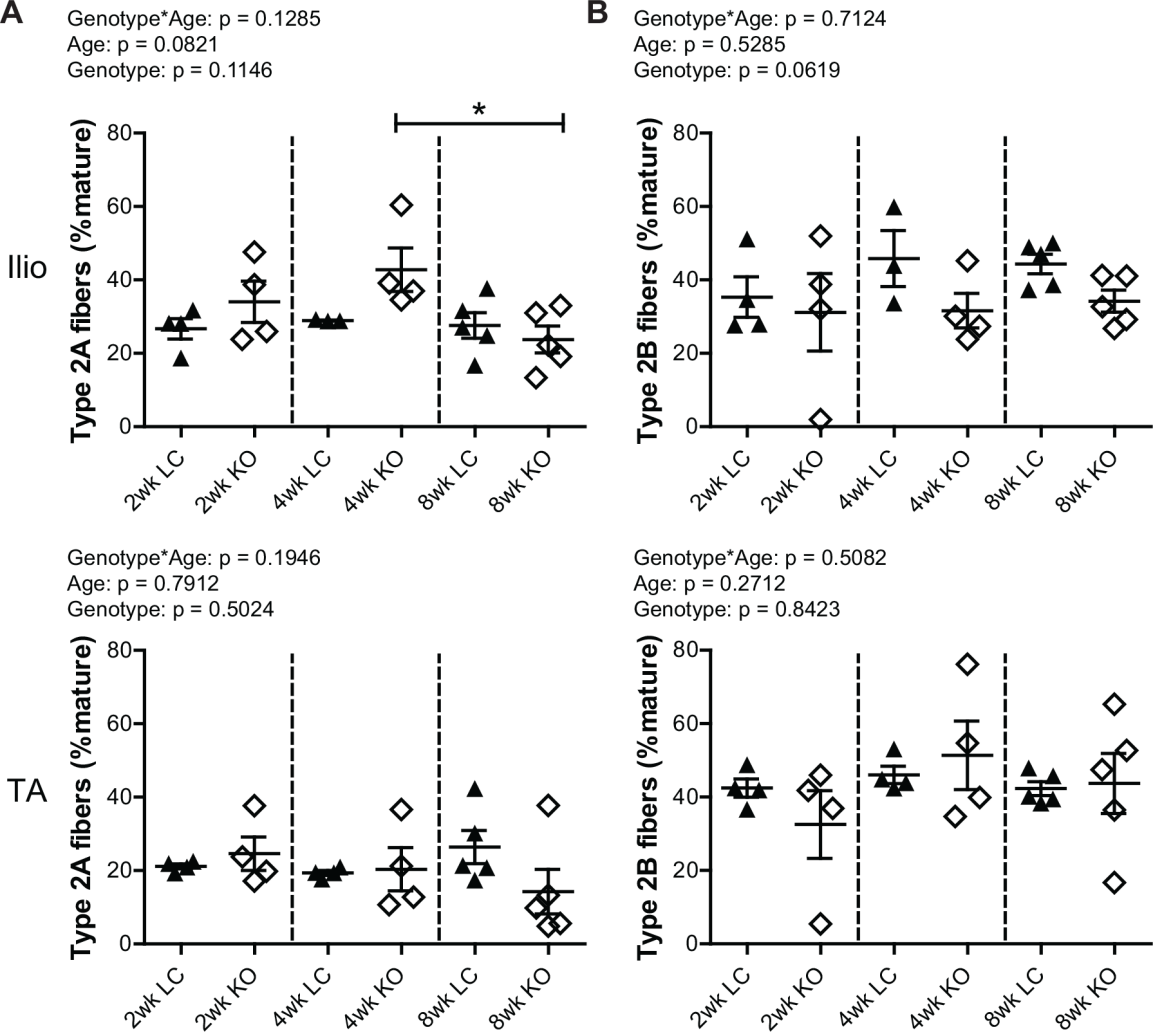
Frequencies of type 2a oxidative and type 2b glycolytic fast-twitch fibers are unchanged in iliopsoas and TA between Myf5/*Fktn* KO and LC mice. (A) Oxidative type 2a fibers in iliopsoas (top) and TA (bottom) of 2, 4, and 8 wko Myf5/*Fktn*-deficient (KO) and control (LC) mice. (B) Glycolytic type 2b fibers in iliopsoas (top) and TA (bottom) of 2, 4, and 8 wko Myf5/*Fktn-*deficient (KO) and control (LC) mice. *, p<0.05; two-way ANOVA with Bonferroni’s post-test. n = 4 for all 2 and 4 wko experimental groups (except Ilio 4 wko LC, n = 3); n = 5 per 8 wko group.

**Fig 4:**
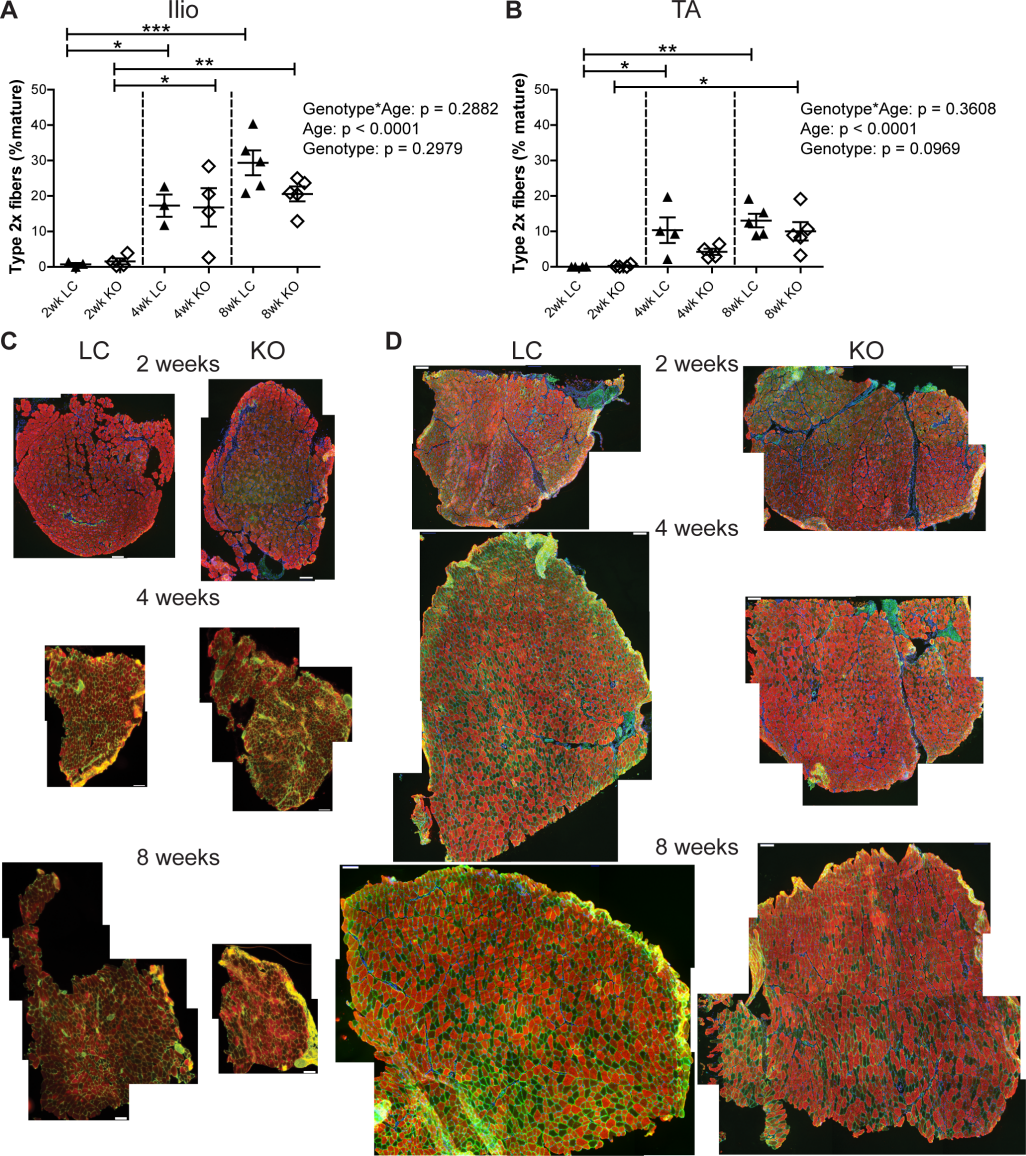
Minor delay in progression of glycolytic type 2x fiber switching in TA of Myf5/*Fktn* KO mice normalizes by 8 weeks. Glycolytic intermediate-twitch 2x fibers in Myf5/*Fktn*-deficient (KO) and control (LC) (A) iliopsoas and (B) TA muscles. *, p<0.05; **, p<0.01; ***, p<0.001, two-way ANOVA with Bonferroni’s post-test. Whole tissue (C) iliopsoas and (D) TA maps of sections stained with antibody detecting all myosin isoforms except type 2x (red), with basement membrane perlecan or sarcolemmal αDG core protein (green) and nuclear (blue) counterstains. Unstained (negative) fibers were counted to measure type 2x. Scale bar = 100μm. n = 4 for all 2 and 4 wko measurements (except Ilio 4wko LC, n = 3); n = 5 per 8 wko group.

To assess whether *Fktn* loss alters muscle fiber differentiation, iliopsoas and TA muscles from 2, 4, and 8 wko Myf5/*Fktn* KO and LC mice were analyzed for expression of mature myosin heavy chain isoforms, indicative of different muscle fiber types. The iliopsoas was chosen because it is a smaller, proximal muscle that is more severely affected in Myf5/*Fktn* KO mice, while the TA is widely used in physiological assessment of muscle. In order to account for differences in the numbers of fibers in muscles across the sample set, data were analyzed as the proportion of isotype positive fibers per total mature fibers counted (presented here as percentages). By this measurement, there was no interaction between genotype and age (genotype*age) and no genotype effect for either iliopsoas or TA in type 1 (oxidative, slow-twitch) fibers, but there was a significant age effect in both muscles with fewer type 1 fibers in older mice compared to younger mice (Fig 2). In post-test analysis, type 1 fibers significantly decreased in iliopsoas of Myf5/*Fktn* KO from 4 to 8 weeks (Fig 2A and 2C), whereas in the TA, type 1 fibers were significantly decreased in all mice from 2 to 8 wko and in Myf5/*Fktn* KO mice from 4 to 8 wko, indicating that postnatal depletion of the type 1 fiber pool may proceed more slowly in Myf5/*Fktn* KO mice (Fig 2B and 2D). Neither type 2a (fast oxidative) nor type 2b (fast glycolytic) fibers were changed in the iliopsoas or TA muscles of Myf5/*Fktn* KOs compared to LCs at any time points examined, although type 2a fibers were reduced in 8 wko compared to 4 wko KO mice (Fig 3). The proportion of type 2x (intermediate glycolytic) fibers was significantly affected by age, but not genotype nor genotype*age interaction (Fig 4). Type 2x were not yet developed in 2 wko mice but progressively increased through 8 weeks of age in iliopsoas and TA muscles. In post-test analysis, both 4 and 8 wko mice had more type 2x fibers in the iliopsoas compared to 2 wko mice, while in the TA, Myf5/*Fktn* LC had increased type 2x fibers at 4 and 8 weeks, but KO mice type 2x fibers were only elevated at 8 weeks (Fig 4). Thus, the transition timing to widespread type 2x fiber specification in TA may be minimally affected in *Fktn*-deficient muscle. Altogether these data demonstrate that postnatal mature fiber type specification is primarily age-dependent, with minimal or no affect due to *Fktn-*deficiency.

### Muscle fiber differentiation is altered following induced regeneration in post-development, whole-body compared to developmental, muscle-specific *Fktn* KO mice

To understand the role of functionally glycosylated αDG in fiber type specification of regenerated fibers, we used cardiotoxin (CTX) to induce widespread, synchronized regeneration in two different *Fktn*-deficient mouse models: developmental, skeletal muscle-specific Myf5/*Fktn* KO (as above) and whole animal, Cre-ER *Fktn* mice, in which *Fktn* deletion is induced by tamoxifen-mediated translocation of Cre-ER to the nucleus for gene excision (Tam/*Fktn* KO are Tam-treated Tg^+/Cre-ER^,Fktn^L/-^; and Tam/*Fktn* LC mice are Tam-treated Tg^+/Cre-ER^,Fktn^L/+^, Tg^+/+^,Fktn^L/-^, or Tg^+/+^,Fktn^L/+^) [15]. The tibialis anterior (TA) was chosen for analysis because it is the smallest muscle that is easily accessible for intramuscular CTX delivery, ensuring that a greater proportion of the muscle compartment is toxin affected. Myf5/*Fktn* mice were toxin injected at 6 wko and muscle was collected 2 weeks after injury at 8 wko. As Myf5 expression begins at embryonic day 8 [31,32], mouse gestation is ~19 days, and tissue was collected at 8 wko, the Myf5/*Fktn* KO muscle is approximately 10 weeks post-*Fktn* deletion. Therefore, Tam/*Fktn* mice were sacrificed 10 weeks following tamoxifen induction of *Fktn* deletion so that the length of *Fktn*-deficiency was comparable between Tam/*Fktn* and Myf5/*Fktn* KO animals. Loss of *Fktn* function was confirmed by staining TA sections with the αDG functional glycan specific antibody IIH6. In saline injected TA, the vast majority of fibers in both Myf5/*Fktn* and Tam/*Fktn* KOs were negative for glycosylated αDG; however, Tam/*Fktn* KOs had significantly higher residual IIH6 levels than their Myf5/*Fktn* counterparts (S1 Fig). As the efficiency of gene knockout in muscle satellite cells of Tam/*Fktn* KOs has not been previously addressed and because Myf5/*Fktn* KOs exhibit minor fiber populations with residual αDG glycosylation, we also examined IIH6 staining in Myf5/*Fktn* and Tam/*Fktn* KOs 14 days after toxin injection. We observed no differences in the proportions of IIH6-positive fibers between saline- and toxin-injected TAs in either mouse strain (n = 6), indicating that regenerated fibers are not disproportionately arising from cell populations that may have escaped *Fktn* gene excision (S1 Fig).

**Fig 5:**
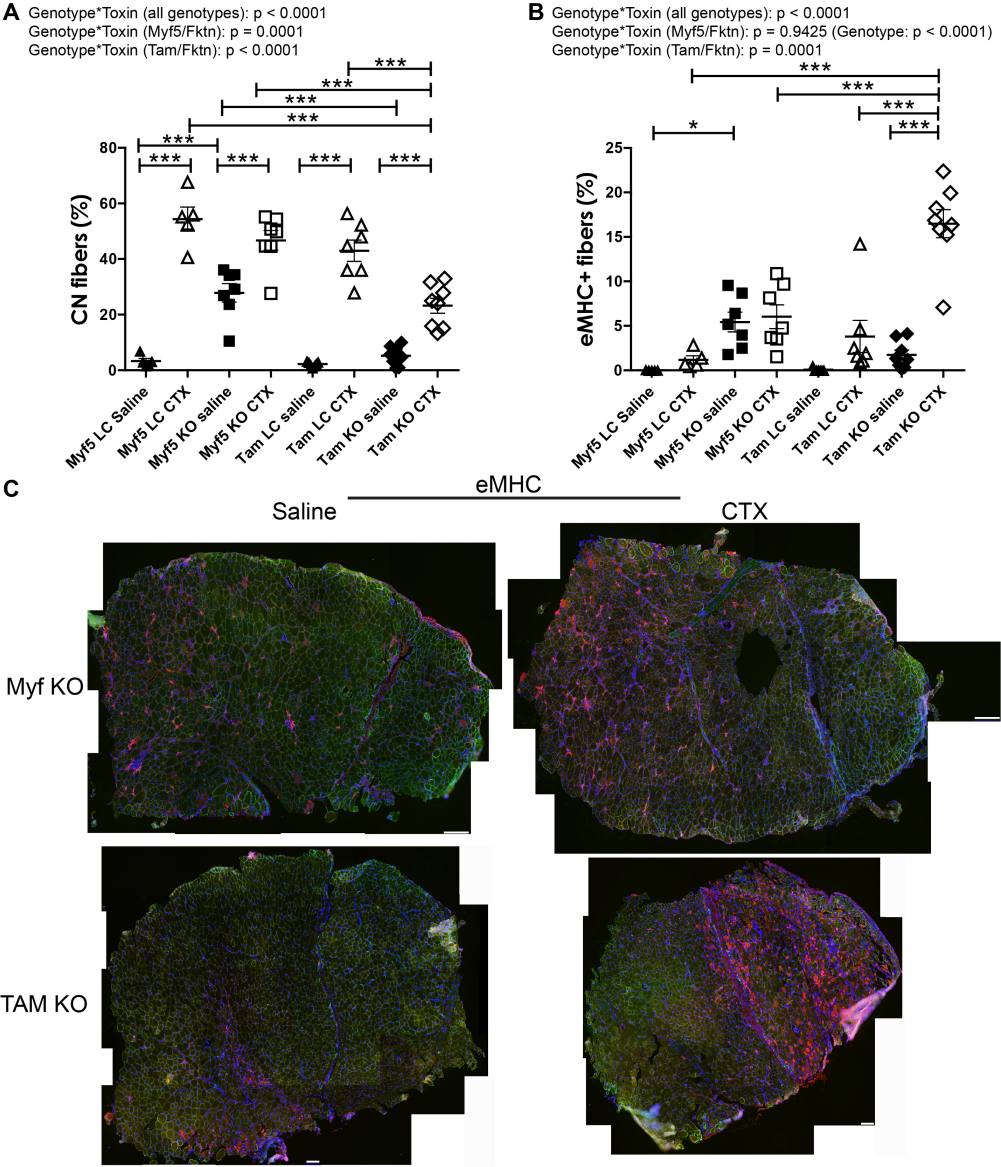
Differentiation is delayed in whole-body Tam/*Fktn* KO mice following cardiotoxin (CTX) induced regeneration. Muscle regeneration measured by long- (CN, (A)) or short-term (eMHC, (B)) markers in muscle specific (Myf5) and whole-body (Tam) *Fktn* KO and LC mice following intramuscular injection with saline or CTX. *, p<0.05; **, p<0.01; ***, p<0.001; two-way ANOVA with Bonferroni’s post-test (all genotypes combined) depicted on figures; two-way ANOVA per strain (Myf5/*Fktn* or Tam/*Fktn*) are also reported. (C) Whole tissue maps of tibialis anterior (TA) muscles from Myf5/*Fktn* or Tam/*Fktn* KO muscle injected with saline or CTX stained with anti-eMHC antibody (red), with sarcolemmal αDG core protein (green) and nuclear (blue) counterstains. Scale bar = 100 μm. n = 5, Myf5 LC; n = 7, Myf5 KO and Tam LC; n = 8, Tam KO.

In analysis of regeneration 14 days post-toxin injury, there was a significant interaction between genotype and toxin (genotype*toxin) for CN and eMHC measures of skeletal muscle regeneration (Fig 5). In post-hoc analysis, all CTX-treated groups had higher CN compared to saline (contralateral) controls (Fig 5A; saline-treated CN means range 2.3 ± 0.3% [Tam LC] to 27.8 ± 3.3% [Myf5 KO] vs. toxin-treated CN means range 23.2 ± 2.7% [Tam KO] to 54.4 ± 4.3% [Myf5 LC]). CN was also elevated in saline Myf5/*Fktn* KO compared to saline Myf5/*Fktn* LC TA muscle, similar to findings in naïve 8 wko TA and iliopsoas muscles (Fig 1, 5A). In contrast, CN was not different between saline-injected Tam/*Fktn* KO and LC mice and was significantly lower in saline-injected Tam/*Fktn* KO than in Myf5/*Fktn* KO muscle (Fig 5A and 5C). Interestingly, proportions of CN fibers were significantly lower 14 days post-CTX in Tam/*Fktn* KO mice compared to all other CTX-treated groups, suggesting that a potential defect or delay in regeneration/differentiation accompanies whole-body *Fktn* excision (Fig 5A and 5C). This interpretation is supported by the unexpected finding that eMHC in Tam/*Fktn* KO CTX muscle was significantly increased relative to both Tam/*Fktn* KO saline muscle and all other CTX groups 14 days after treatment (Fig 5B and 5C; Tam KO eMHC = 16.5 ± 1.6%; all other groups, mean eMHCs range from 0.0 ± 0.0 to 6.0 ± 1.3%). We previously noted that eMHC positive fiber areas were much smaller in naïve 20 week old Myf5/*Fktn* KO compared to a milder, mature-muscle specific knockout MCK/*Fktn* KO mice and in Myf5/*Fktn* KO versus LC 7 days post toxin-injury [15]; therefore, we analyzed the size of eMHC expressing fibers in the 14 day post-toxin Myf5/*Fktn* and Tam/*Fktn* KO mice here. At 14 days post-toxin, regenerating (eMHC+) fibers tended to be very small in both Myf5/*Fktn* and Tam/*Fktn* TAs. However, Myf5/*Fktn* KOs had substantially fewer transiently regenerating fibers at this time point (55–366 fibers, n = 7) compared to their Tam/*Fktn* counterparts (337–1840 fibers, n = 8)(S2 Fig). Note, very small eMHC expressing fibers generally do not have central nuclei, so the reduction in Tam/*Fktn* KO CN can be explained, in part, but the high number of small eMHC remaining at 14 days post-toxin in this model. These data suggest that either αDG function in regeneration is dependent on its presence or absence during development or that non-muscle αDG, such as brain or motor neuron αDG, is also critical for regeneration or the transition to terminal differentiation in muscle fibers post-injury.

**Fig 6:**
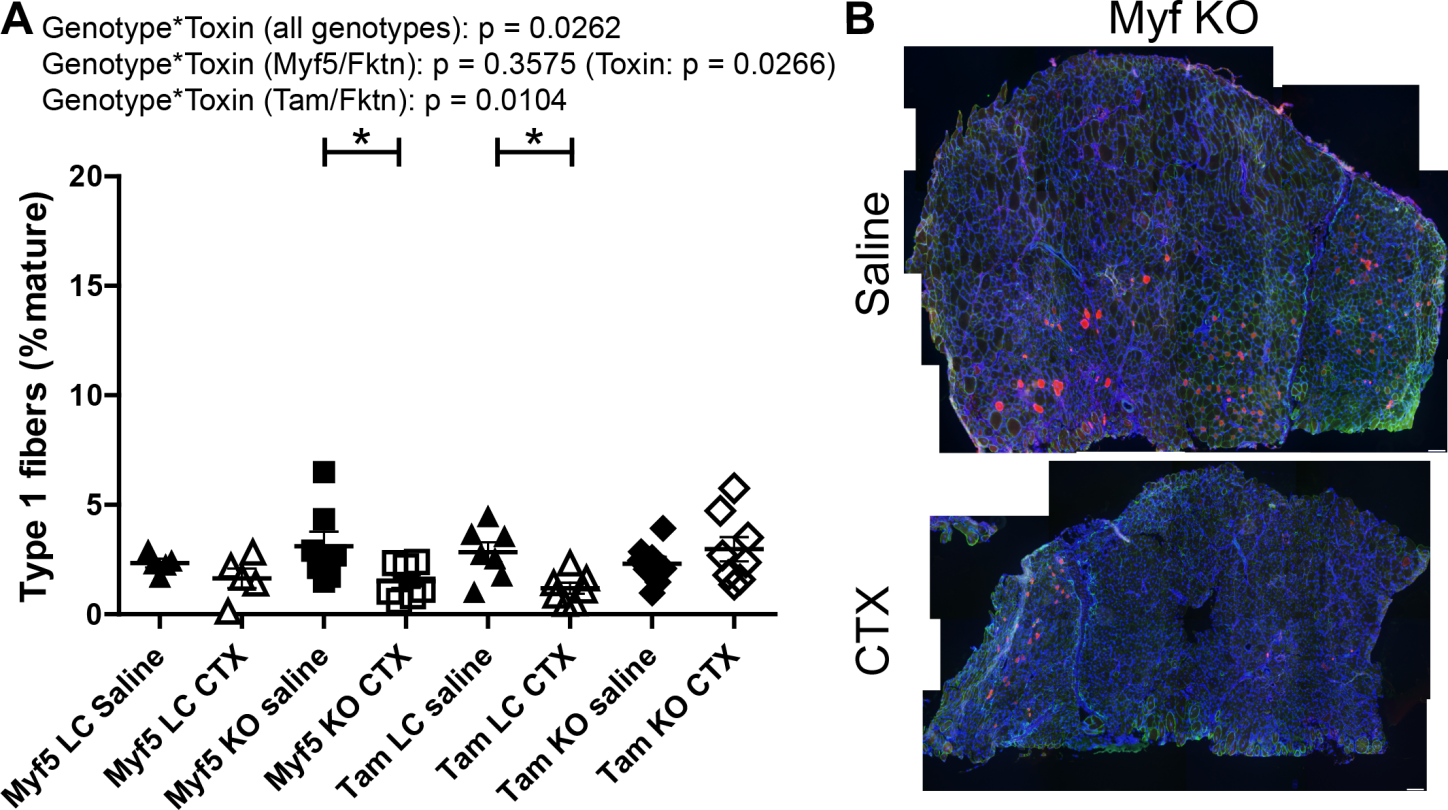
Slow oxidative fibers are decreased in Myf5/*Fktn* KO and Tam/*Fktn* LC mice following CTX injection. (A) Quantification of slow type 1 oxidative fibers in TA muscle of saline- or CTX-injected Myf5/*Fktn* and Tam/*Fktn* LC or KO mice. *, p<0.05; **, p<0.01; ***, p < .001; two-way ANOVA with Bonferroni’s post-test (all genotypes combined) depicted on figures; two-way ANOVA per strain (Myf5/*Fktn* or Tam/*Fktn*) are also reported. (B) Whole tissue maps of CTX-injected TA muscle stained with anti-myosin heavy chain type 1 antibody (red), with sarcolemmal αDG core protein (green) and nuclear (blue) counterstains. Scale bar = 100 μm. n = 5, Myf5 LC; n = 7, Myf5 KO and Tam LC; n = 8, Tam KO.

**Fig 7:**
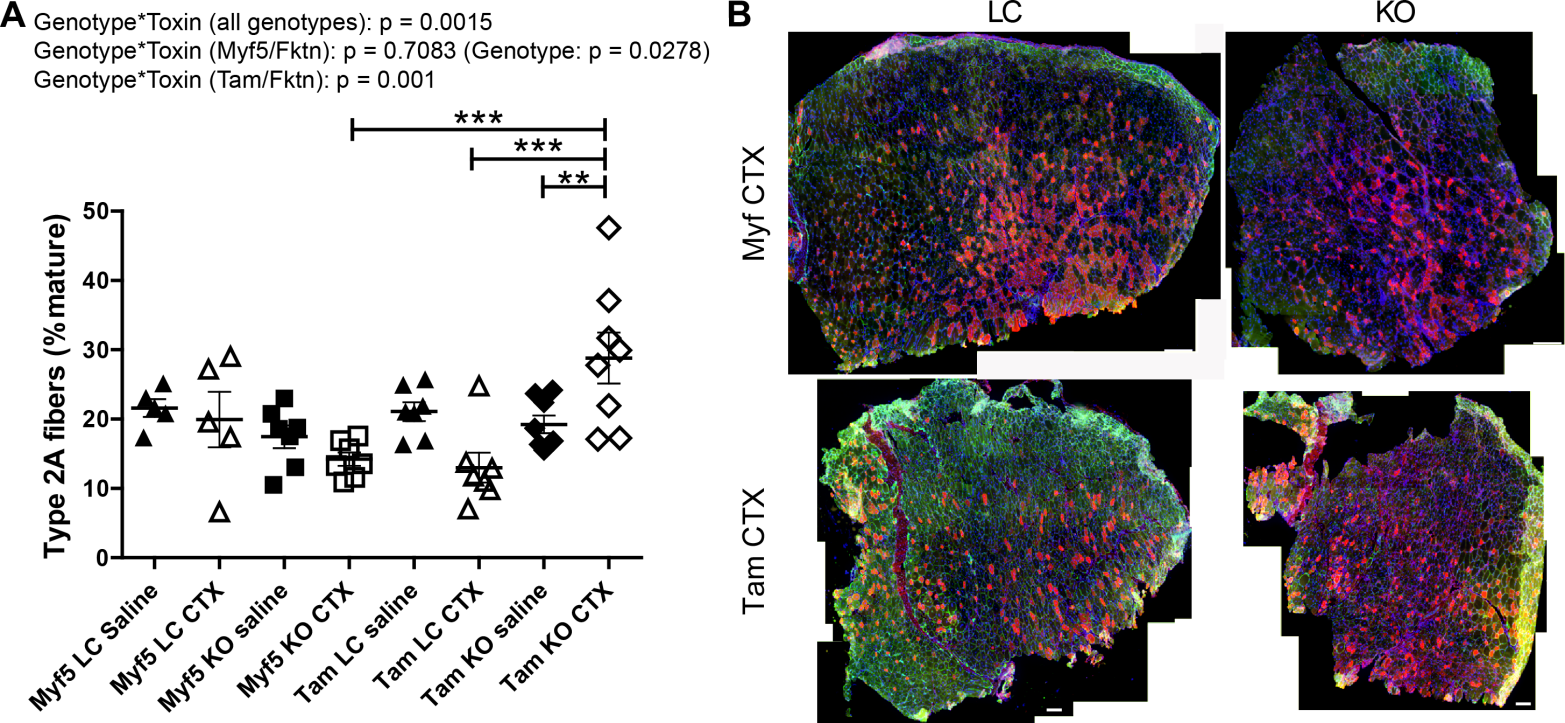
Fast oxidative fibers are increased in Tam/*Fktn* KO mice following CTX injection. (A) Quantification of fast type 2a oxidative fibers in TA muscle of saline- or CTX-injected Myf5/*Fktn* and Tam/*Fktn* LC or KO mice. *, p<0.05; **, p<0.01; ***, p < .001; two-way ANOVA with Bonferroni’s post-test (all genotypes combined) depicted on figures; two-way ANOVA per strain (Myf5/*Fktn* or Tam/*Fktn*) are also reported. (B) Whole tissue maps of CTX-injected TA muscle stained with anti-myosin heavy chain type 2a antibody (red), with sarcolemmal αDG core protein (green) and nuclear (blue) counterstains. Scale bar = 200 μm. n = 5, Myf5 LC; n = 7, Myf5 KO and Tam LC; n = 8, Tam KO.

In order to clarify the role of αDG glycosylation in muscle fiber type specification, we examined fiber type distributions in TA muscle of saline- or toxin-injected Tam/*Fktn* and Myf5/*Fktn* mice. There was a genotype*toxin interaction across all genotypes for proportions of type 1 (slow-oxidative) fibers due primarily to a genotype*toxin interaction in Tam/*Fktn* mice (Fig 6). Type 2a (fast-oxidative) fibers were subject to genotype*toxin interaction, primarily due to different toxin-induced regenerative responses between Myf5/*Fktn* KO and Tam/*Fktn* KO as detected by statistical post-tests. However, a genotype*toxin interaction and a genotype group effect were detected in independent analyses of Tam/*Fktn* and Myf5/*Fktn* strains, respectively (Fig 7), further indicating the possibility of abnormal differentiation of regenerated fibers in whole-body *Fktn* KOs.

**Fig 8:**
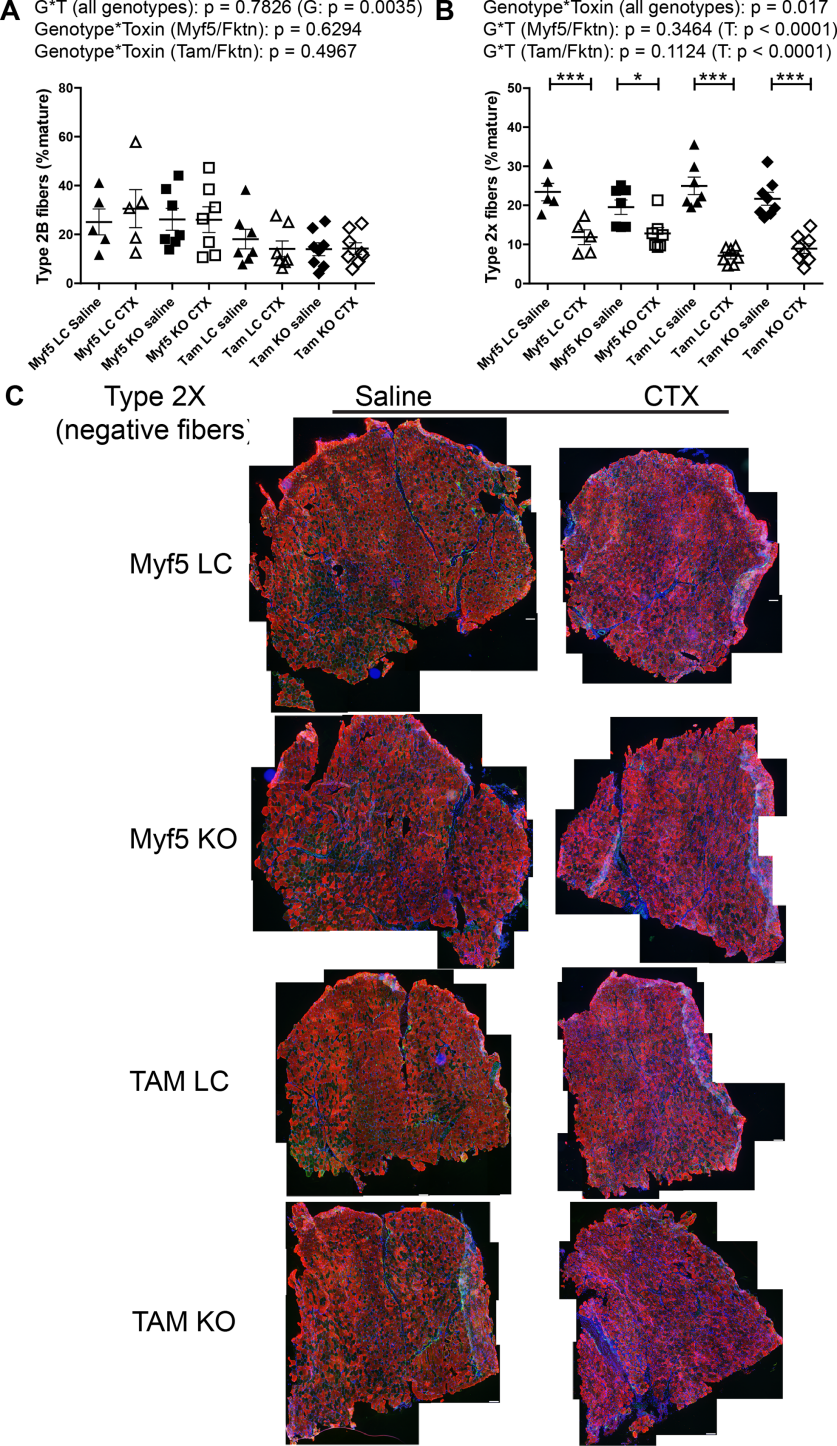
Glycolytic type 2x, but not type 2b, fibers decrease following muscle regeneration. Glycolytic type 2b (A) or type 2x (B) fiber proportions in muscle-specific (Myf5) or whole-body (Tam) *Fktn* KO and LC muscle injected with saline or CTX. *, p<0.05; ***, p<0.001; two-way ANOVA with Bonferroni’s post-test (all genotypes combined) depicted on figures; two-way ANOVA per strain (Myf5/*Fktn* or Tam/*Fktn*) are also reported. (C) Whole tissue maps of TA muscle from Myf5/*Fktn* or Tam/*Fktn* KO and LC muscle injected with saline or CTX and stained with an antibody detecting all myosin heavy chain isoforms except type 2x. Unstained (negative) fibers were counted to measure type 2x. Scale bar = 100 μm. n = 5, Myf5 LC; n = 7, Myf5 KO and Tam LC; n = 8, Tam KO.

**Fig 9:**
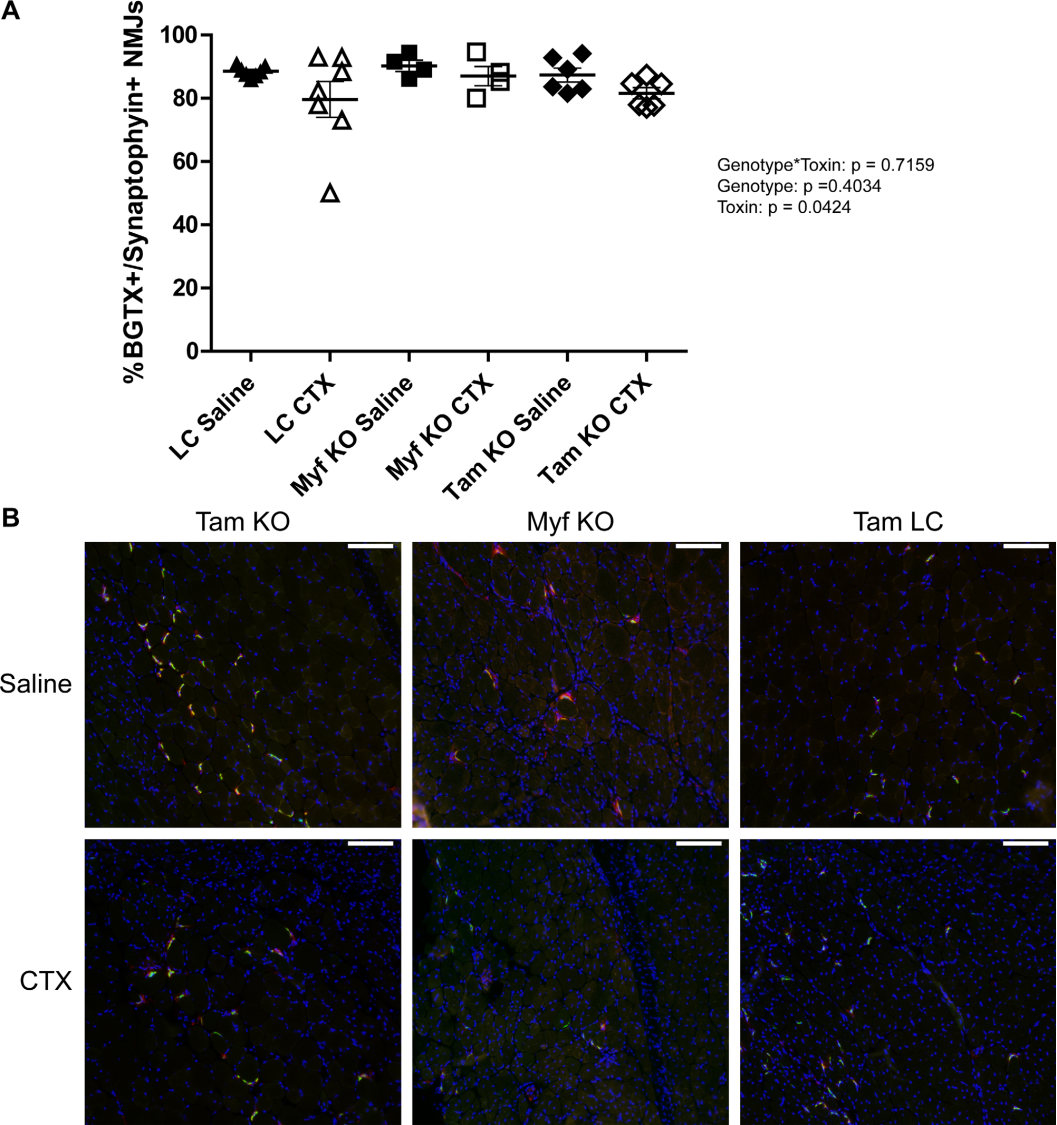
Both presynaptic and postsynaptic components are present at neuromuscular junctions in 14 d regenerated muscle of Myf5/*Fktn* and Tam/*Fktn* KOs. (A) Proportion of NMJs positive for synaptophysin and BGTX in saline- or CTX-injected TA of Myf5/*Fktn* and Tam/*Fktn* KOs or grouped Myf5/Tam LCs. (B) Representative images showing localization of NMJ pre-synaptic (synaptophysin, red) and post-synaptic markers (BGTX, green) in Myf5/*Fktn* KO and Tam/*Fktn* KO and LC TAs injected with saline or CTX. Nuclei stained with DAPI (blue). Scale bar = 100μm. n = 7, LC; n = 4, Myf5 KO; n = 6 Tam KO.

Analysis of type 2b fibers revealed substantial variability in the prevalence of this fiber type across all groups; however, there was a significant genotype effect as all Tam/*Fktn* mice had lower means for type 2b than Myf5/*Fktn* mice (including littermates), which may reflect an age dependent type 2b fiber preference between the two strains in the study, with no effect of *Fktn* deficiency (Fig 8A). In contrast, a genotype*toxin interaction was detected for type 2x fibers. In post-test comparisons, regeneration after CTX injury decreased the relative frequency of type 2x fibers in all groups (Fig 8). The presence of a toxin-induced regeneration effect, but no interaction in independent analyses of Myf5/*Fktn* and Tam/*Fktn* strains, suggests that the overall genotype*toxin effect (all genotypes) reflects the greater magnitude of type 2x fiber loss in the Tam/*Fktn* cohort compared to the Myf5/*Fktn* cohort, possibly an age rather than a knockout effect. In sum, these results indicate specific roles for functional αDG in the transition from eMHC and in the differentiation to type 1 and 2a fibers in regenerated skeletal muscle after injury; however, the contributions of αDG glycosylation in muscle development versus αDG glycosylation in non-muscle tissues are not fully clear. In particular, loss of *Fktn* in pre-synaptic motor neurons of the neuromuscular junction (NMJ) in Tam/*Fktn*, but not Myf5/*Fktn*, KO mice could affect muscle innervation and regeneration. Therefore, we probed sections from saline- or toxin-injected TAs of Myf5/*Fktn* and Tam/*Fktn* mice with synaptophysin (for detection of the pre-synaptic component of the NMJ) and α-bungarotoxin (BGTX, for detection of nicotinic acetylcholine receptors at the muscle endplate). While full elaboration of the neuromuscular junction morphology cannot be determined from muscle transverse sections, we did measure NMJ occupancy (colocalization of both presynaptic synaptophysin and postsynaptic BGTX). There was no difference in the innervation/occupancy status of NMJs according to genotype*toxin interaction or genotype, although there was a significant toxin effect, apparently due to increased variability and a reduced group mean in injured littermate mice (Fig 9).

## Discussion

Skeletal muscles exhibit fiber type mosaicism, but myofibrillar phenotype varies considerably between muscles according to muscle use. A growing body of work has shown shifts in fiber type predomination of muscles in various dystrophic conditions, but the underlying mechanism of these changes is not fully understood [22,24,33–35]. Decreased muscle function in dystrophic patients and mouse models often involves components of both muscle weakness and fatigue, so enhanced populations of low-endurance, fast-twitch fiber types may negatively affect performance in dystrophic tissues [36,37], suggesting a protective role for activation of the oxidative myogenic program. However, these observations do not extend to all muscles, as the vastus lateralis of both human DMD patients and GRMD dogs show a reduction of type 2 fibers, suggesting that enhancement oxidative program may actually be deleterious in some muscles [38–40].

Despite extensive examination of fiber types in dystrophin-deficient muscle, little work has been done in models of aberrant αDG glycosylation. Analysis of biopsies from rectus femoris or biceps brachii of Fukuyama-type congenital muscular dystrophy (FCMD) patients, whose muscles contain defective *Fktn*, revealed increased type 1 and decreased type 2b fibers in a subset of patients; however, differences in the ages of the patients sampled may have contributed to inter-patient variability with respect to muscle fiber types [41]. A study in the *LARGE*^myd/myd^ mouse, a spontaneous mutation model for dystroglycanopathy, found that while both the soleus and EDL muscles were weak in myd mice, the soleus was resistant to force loss following lengthening contraction [26]. The soleus had increased β1 integrin, suggesting an increase in the frequency of the other major laminin-binding complex in muscle, α7β1 integrin. However, a clear relationship between fiber type and integrin expression has not been established and it is therefore unclear whether oxidative fiber types, increased integrin content, or a combination of the two protect the soleus from lengthening-contraction induced damage. The low proportion of type 1 fibers in the TA and iliopsoas analyzed here may not reflect fiber dynamics in muscles enriched for slow, oxidative contraction, like the soleus. Overall, it remains unclear if the altered fiber phenotypes identified here help to drive the disease process or are simply biomarkers of underlying pathogenic or nonpathogenic differences due to altered αDG structural or signaling axis.

In spontaneous models of MD, it is difficult to distinguish the roles of the affected gene in developmental, regenerating, or muscle-extrinsic regulation of fiber type. In particular, dystrophic muscle undergoes cycles of early, highly active and later, slowly progressive disease states as the regenerative capacity of the muscle is exhausted [42,43]. To more clearly determine the roles of αDG in muscle regeneration and fiber type specification, we induced widespread regeneration in both muscle-specific (Myf5) and whole body (Tam, inducible) *Fktn* KO muscle. We noted clear differences in the numbers of regenerated fibers (CN) between saline-treated Myf5/*Fktn* and Tam/*Fktn* KO muscle, suggesting that early loss of αDG glycosylation has developmental consequences that may enhance muscle susceptibility to damage. We previously showed that there were more eMHC-positive (regenerating) fibers in 20 week old Myf5/Fktn KO mice compared to a milder, mature muscle MCK/*Fktn* KO model, and that the eMHC-positive fibers were much smaller in both naïve Myf5/*Fktn* versus MCK/*Fktn* KO muscle and in 7 day post-toxin Myf5/*Fktn* KO versus LC muscle. As expected, the proportion of very small regenerating fibers (< 75μm^2^) was lower at 14 days in this study versus 7 days post-toxin in Myf5/*Fktn* KOs (~35% 14 days post to ~55% 7 days post) previously reported (15), indicating further growth and maturation of the regenerated muscle fiber pool with the additional time post injury. However, we report the surprising finding that post-development, whole-body inducible Tam/*Fktn* KOs had significantly more eMHC-positive fibers compared to all other toxin injected groups; furthermore, regenerating fibers in Tam/*Fktn* KOs were significantly smaller than those in Myf5/*Fktn* KOs. Notably, delayed muscle fiber maturation has also been reported in FCMD muscles [44]. Thus, while developmental, muscle-specific *Fktn* ablation appears to increase vulnerability to damage, post-developmental, muscle-intrinsic and -extrinsic *Fktn* loss may also lead to defective and/or delayed regeneration. Specifically, differences between Myf5/*Fktn* and Tam/*Fktn* KO mice could be due to the presence/absence of glycosylated αDG in muscle development, the presence/absence of glycosylated αDG in motor neurons, and/or mouse age.

Motor neurons play a significant role in post-developmental fiber specification, but do not appear to dictate fiber type in developing muscle; furthermore, muscle innervation is at least partially responsible for the induction of the slow-fiber program, meaning that the developmental muscle-intrinsic fiber-specification pathway, under normal conditions, favors fast-type fibers [19,45,46]. Interestingly, we found that CTX-induced regeneration promoted an increase in the oxidative phenotype in whole-body Tam/*Fktn* KO mice but no change or a decrease in the oxidative phenotype in early Myf5/*Fktn* KO mice. Together, these results suggest that functionally glycosylated αDG may be important in both the presynaptic motor neuron (muscle extrinsic) and postsynaptic muscle (muscle intrinsic) components of muscle regeneration and fiber type determination or that developmental loss of αDG processing impacts the determination of muscle regeneration and subsequent muscle fiber type post-development. Since muscle from both Myf5/*Fktn* and Tam/*Fktn* KO mice are *Fktn*-deficient in skeletal muscle myofibers and satellite cells, it is expected that the muscle intrinsic sensory signaling pathways controlling fiber specification should be comparable. Thus, *Fktn* loss in motor neurons may affect myosin expression through altered activity, although NMJ occupancy was not different between LC and KO mice. However, there is also evidence that specific myoblast populations formed during development, may favor generation of different fiber types; specifically, satellite cell subpopulations tend to form muscle of predetermined fiber types [19,47,48]. Since *Fktn* deletion is initiated at E8 in Myf5/*Fktn* KO mice, muscle progenitors in developing muscle are affected by hypoglycosylation of αDG, representing an alternative explanation for phenotypic differences between Myf5/*Fktn* and Tam/*Fktn* KO mice. It is likely that a combination of αDG-driven developmental and neuronal factors contribute to fiber type specification; however, additional work will be required to clarify such mechanisms.

## Supporting Information

S1 Fig Glycosylated αDG-positive fibers do not increase in TAM/Fktn KO mice after toxin injection. TA muscle sections showing glycosylated αDG (antibody IIH6, red) and core αDG protein (antibody 5–2, green) with DAPI nuclear counterstain 14 days post-saline or cardiotoxin (CTX). Scale bar, 200μm. White arrows mark some representative fibers with glycosylated αDG. Note, red punctate staining is background from mouse IgM-A546 secondary antibody.
(TIF)

S2 Fig Regenerating fibers are smaller in Tam/*Fktn* versus Myf5/*Fktn* KOs 14 days post-injury. Proportions of eMHC-expressing muscle fibers in TAs of Myf5/*Fktn* (black bars) or Tam/*Fktn* (white bars) KO mice 14 days after CTX injection grouped according to size. The number of eMHC-positive muscle fibers ranged from 55–366 in Myf5/*Fktn* (n = 7) and 337–1840 in Tam/*Fktn* KOs (n = 8). p < .0001, two-tailed Mann-Whitney test.
(TIF)
