# Supplementary figures and images for: Abnormal Skeletal Muscle Regeneration plus Mild Alterations in Mature Fiber Type Specification in Fktn-Deficient Dystroglycanopathy Muscular Dystrophy Mice
Source: PLoS One. 2016 Jan 11;11(1):e0147049. doi: 10.1371/journal.pone.0147049 (PMC4708996; doi:10.1371/journal.pone.0147049)

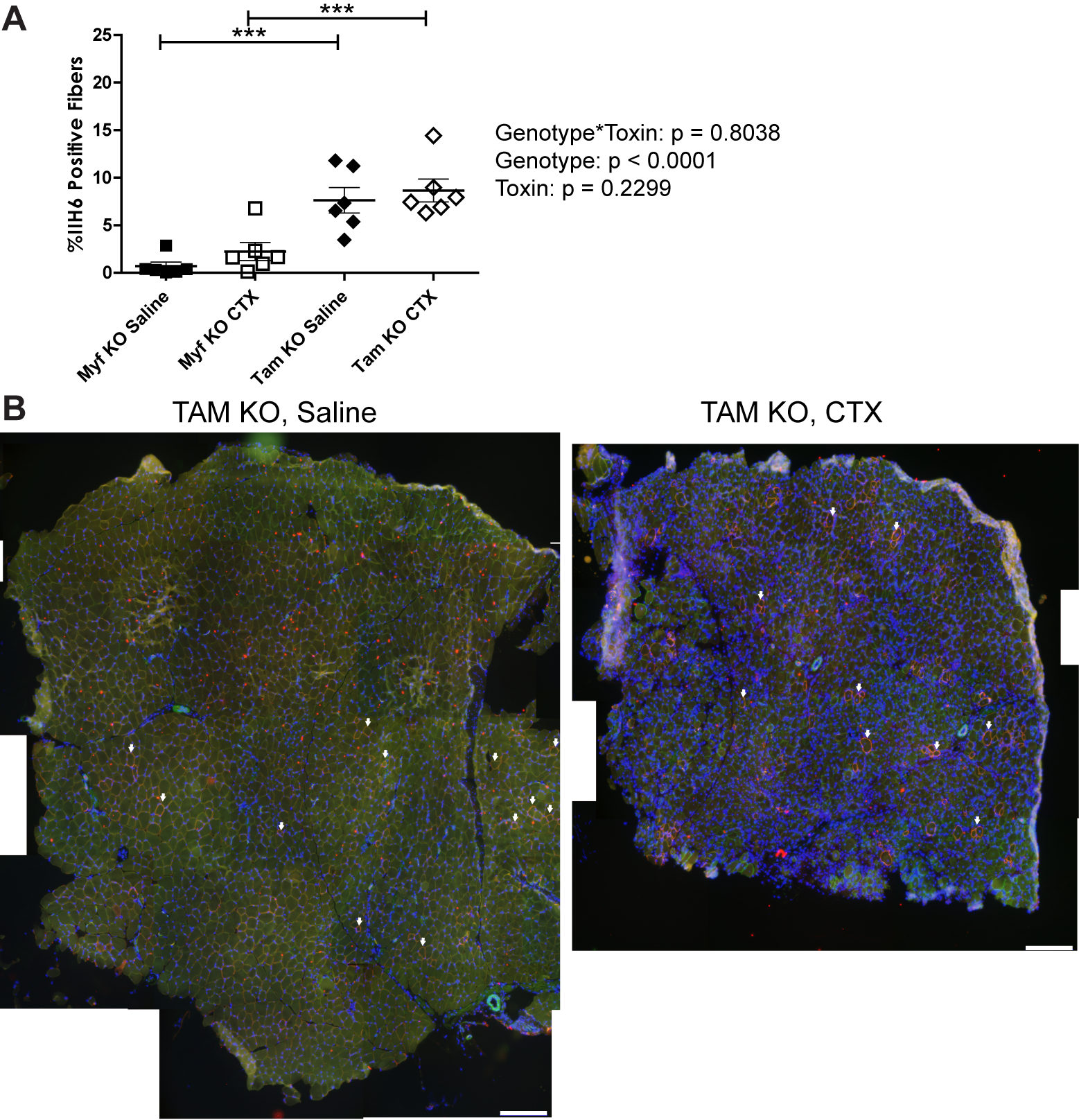

Supplement: S1 Fig — TA muscle sections showing glycosylated αDG (antibody IIH6, red) and core αDG protein (antibody 5–2, green) with DAPI nuclear counterstain 14 days post-saline or cardiotoxin (CTX). Scale bar, 200μm. White arrows mark some representative fibers with glycosylated αDG. Note, red punctate staining is background from mouse IgM-A546 secondary antibody. (TIF) [file pone.0147049.s001.tif]

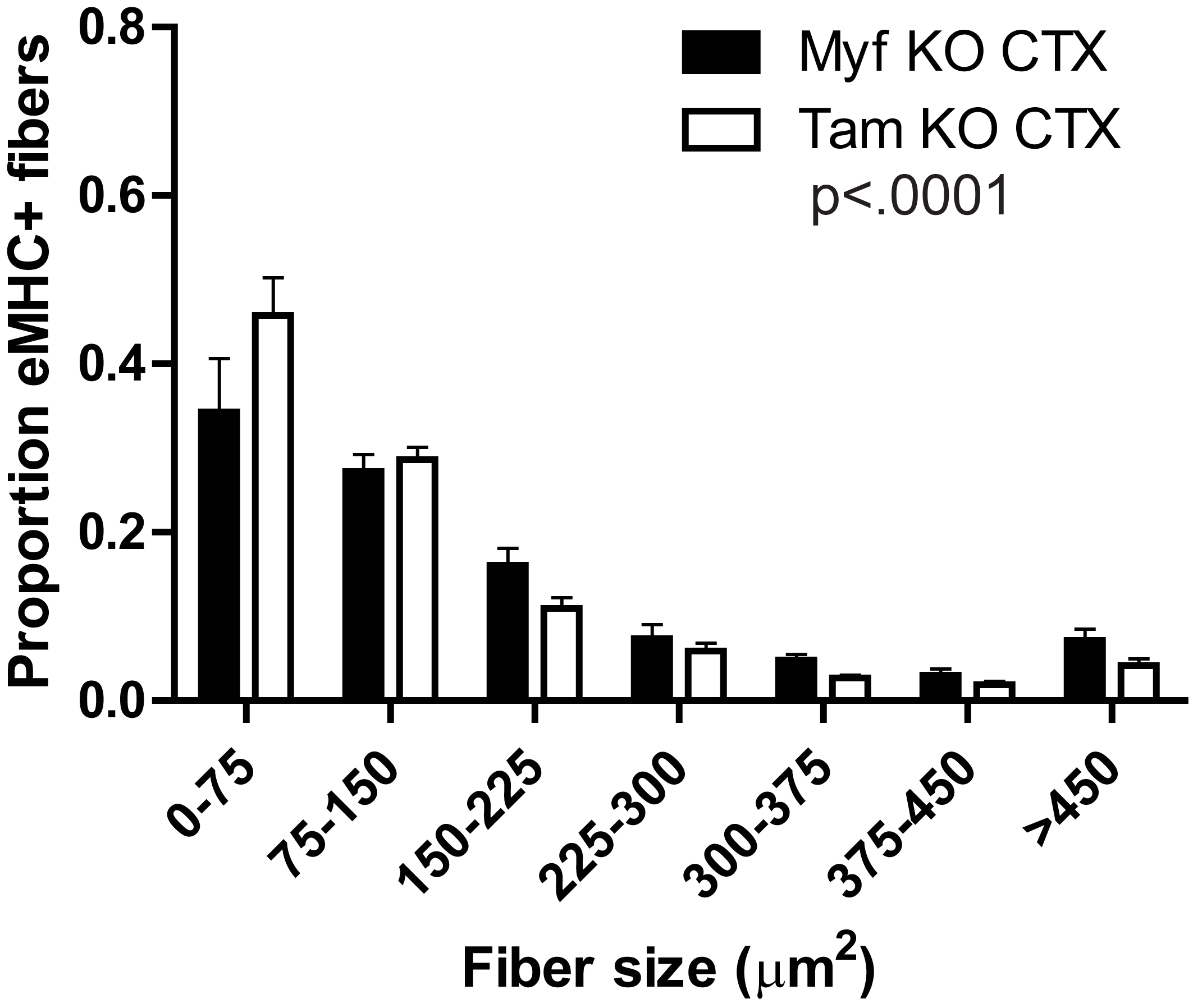

Supplement: S2 Fig — Proportions of eMHC-expressing muscle fibers in TAs of Myf5/Fktn (black bars) or Tam/Fktn (white bars) KO mice 14 days after CTX injection grouped according to size. The number of eMHC-positive muscle fibers ranged from 55–366 in Myf5/Fktn (n = 7) and 337–1840 in Tam/Fktn KOs (n = 8). p < .0001, two-tailed Mann-Whitney test. (TIF) [file pone.0147049.s002.tif]
